# Pre-existing virus-specific CD8^+^ T-cells provide protection against pneumovirus-induced disease in mice

**DOI:** 10.1016/j.vaccine.2012.08.027

**Published:** 2012-10-05

**Authors:** Mary J.G. van Helden, Peter J.S. van Kooten, Cornelis P.J. Bekker, Andrea Gröne, David J. Topham, Andrew J. Easton, Claire J.P. Boog, Dirk H. Busch, Dietmar M.W. Zaiss, Alice J.A.M. Sijts

**Affiliations:** a Division of Immunology, Faculty of Veterinary Medicine, University of Utrecht, Yalelaan 1, 3584 CL Utrecht, The Netherlands; b Division of Pathology, Faculty of Veterinary Medicine, University of Utrecht, Yalelaan 1, 3584 CL Utrecht, The Netherlands; c D. Smith Center for Vaccine Biology and Immunology, University of Rochester Medical Center, 601 Elmwood Avenue, Rochester, NY 14642, USA; d School of Life Sciences, Gibbet Hill Campus, University of Warwick, Coventry CV4 7AL, UK; e Department of Vaccinology, Centre for Infectious Disease Control, National Institute for Public Health and the Environment (RIVM), Antonie van Leeuwenhoeklaan 9, 3721 MA Bilthoven, The Netherlands; f Institute for Medical Microbiology, Immunology and Hygiene, Technische Universität München, Trogerstrasse 30, 81675 München, Germany

**Keywords:** BAL, bronchoalveolar lavage, BALF, BAL fluid, DC, dendritic cell, BM-DC, bone marrow derived DC, DCp, peptide-loaded DC, FI, formalin inactivated, hRSV, human respiratory syncytial virus, ID, infectious dose, EID, egg ID, i.n., intranasal, i.p., intraperitoneal, i.v., intravenous, MLN, mediastinal lymph node, NK, natural killer, NS, nonstructural, p.i., post infection, pfu, plaque forming units, PVM, pneunomia virus of mice, SEM, standard error of mean, Pneumoviruses, Pneunomia virus of mice, NK cell, CD8^+^ T-cell, Vaccine

## Abstract

Pneumoviruses such as pneumonia virus of mice (PVM), bovine respiratory syncytial virus (bRSV) or human (h)RSV are closely related pneumoviruses that cause severe respiratory disease in their respective hosts. It is well-known that T-cell responses are essential in pneumovirus clearance, but pneumovirus-specific T-cell responses also are important mediators of severe immunopathology. In this study we determined whether memory- or pre-existing, transferred virus-specific CD8^+^ T-cells provide protection against PVM-induced disease. We show that during infection with a sublethal dose of PVM, both natural killer (NK) cells and CD8^+^ T-cells expand relatively late. Induction of CD8^+^ T-cell memory against a single CD8^+^ T-cell epitope, by dendritic cell (DC)-peptide immunization, leads to partial protection against PVM challenge and prevents Th2 differentiation of PVM-induced CD4 T-cells. In addition, adoptively transferred PVM-specific CD8^+^ T-cells, covering the entire PVM-specific CD8^+^ T-cell repertoire, provide partial protection from PVM-induced disease. From these data we infer that antigen-specific memory CD8^+^ T-cells offer significant protection to PVM-induced disease. Thus, CD8^+^ T-cells, despite being a major cause of PVM-associated pathology during primary infection, may offer promising targets of a protective pneumovirus vaccine.

## Highlights

► NK cells and CD8^+^ T-cells expand relatively late following pneumovirus infection. ► Memory CD8^+^ T-cells support type 1 skewing of pneumovirus-specific responses. ► Memory CD8^+^ T-cells prevent pneumovirus-induced immunopathology. ► CD8^+^ T-cell targeted immunization protects against pneumovirus-induced disease.

## 1 Introduction

Pneumoviruses are an important cause of respiratory infections in mammals [1]. One well-known member of the pneumovirus genus is hRSV, a major cause of severe respiratory disease in infants and elderly [2]. A failed vaccine trial using formalin-inactivated hRSV (FI-RSV) in the 1960s that led to enhanced disease instead of immune protection [3–6], has triggered intense efforts to elucidate how to induce immune responses that can prevent or protect against natural hRSV infection without causing pathology. Different studies in humans and mouse models have shown that antibodies can contribute to immune protection [7–10]. However, the antibodies induced during natural hRSV infection fail to prevent recurrent infections throughout life, indicating that also the efficacy of vaccine-induced neutralizing antibodies may be limited [7,11]. Controversy also exists concerning the precise role of the T cell compartment in pneumovirus-induced disease [12,13]. Several studies have shown that although T cells are essential in eradicating established infections [14], they also are important mediators of hRSV-induced immunopathology [15–19]. In murine models, especially Th2 skewing of the CD4^+^ T-cell lineage after immunization with FI-RSV or hRSV-G protein encoding recombinant Vaccinia virus vectors have been shown to lead to enhanced disease following subsequent hRSV infection [12,13,20]. Induction of CD8^+^ T-cell responses, on the other hand, inhibited vaccine-enhanced pulmonary disease [21–23]. Thus, despite the notion that T cells play a role in pneumovirus-induced immunopathology, these studies suggest that vaccines designed to induce antipneumoviral CD8^+^ T cell responses may offer an alternative to vaccines targeting the humoral response.

Pneumoviruses display a narrow host range and several species-specific variants have been described [1], adapted for evasion of defense mechanisms in their specific hosts [24,25]. Therefore, instead of hRSV, its mouse-adapted variant PVM is increasingly used to study pneumovirus-specific immune responses and immunopathogenesis in mouse models. PVM and hRSV display a marked genetic similarity and use similar evasion strategies [26–28]. Intranasal (i.n.) administration of a low PVM inoculum results in effective replication and severe respiratory disease in mice, with several hallmarks similar to severe hRSV disease in humans, including severe pulmonary inflammation, edema, and influx of granulocytes [29].

Although extensively studied during hRSV infections in mouse models, only limited studies evaluated T cells in PVM infected mice [30,31]. Frey et al. showed that, like in hRSV-infection, T-cells are essential for viral elimination in PVM-infected mice, but are also important mediators of infection-associated pathology [31]. This observation raises the question of whether a pneumovirus-vaccine that targets CD8^+^ T cell responses would be safe. In this study, we used the PVM mouse model of respiratory infection to determine whether pre-existing virus-specific CD8^+^ T-cells may provide protection against pneumovirus-induced disease.

## 2 Material and methods

### 2.1 Virus stocks, mice and infection

PVM strain J3666 was passaged in mice to retain full pathogenicity and hRSV strain A2 was grown in BSC-1 cells and concentrated as described [32]. For both viruses, plaque assays on BSC-1 cells were performed to determine viral titers. Influenza strains A/HK/x31 (H3N2) and A/PR/8/34 (H1N1) were grown as described [33]. Age-matched 7–10 week old female BALB/c mice were purchased from Charles River, anesthetized with isoflurane and then infected i.n. with 5 × 10^6^ pfu RSV in 50 μl, or with 1 × 10^5^ EID_50_ HKx31 or 150 EID_50_ PR8 in 30 μl PBS as described [33], or with the indicated doses of PVM in 30 μl PBS. All animal experiments were approved by the Committee on Animal Experiments of the University of Utrecht.

### 2.2 Sample preparation

Mice were sacrificed by injection of sodium pentobarbital and bronchoalveolar lavage (BAL) was collected by three times lavage with 1 ml PBS containing 10 μM EDTA. Thereafter, lungs were perfused with PBS, excised, minced and incubated in PBS containing collagenase (2.4 mg/ml; Roche Applied Science) and DNase (1 mg/ml; Roche Applied Science) for 30 min at 37 °C, passed through a cell strainer and lymphocytes were purified using lympholyte-M (Cederlane). For mRNA isolation, the right lung was placed in 1 ml TRIzol (Invitrogen).

### 2.3 Flow cytometry

Fluorochrome-conjugated antibodies were purchased from eBioscience [CD69 (H1.2F3), CD49b (DX5), TCRβ (H57-597), NKp46 (29A1.4), CD62L (MEL-14), IFNy (XMG1.2), CD8 (53-6.7), CD11c (N418), CD19 (MB19-1), CD4 (RM4-5), MHC-II (m5/114.15.2)] or BD Pharmingen [Siglec-F (E50-2440)]. PE-labeled MHC class I tetramers were prepared in collaboration with D. Busch (TU-Muenchen), by refolding H2-K^d^ heavy chains and human β_2_m in the presence of synthetic influenza-derived NP_147–155_ (TYQRTRALV), hRSV M2_82–90_ (SYIGSINNI) or PVM P_261–269_ (CYLTDRARI). Cell surface markers were stained as described [34]. For tetramer stainings, cells were incubated with 1 μg tetramer for 1 h at 4 °C and then stained for surface markers. To measure IFNγ production, BAL cells were stimulated 1:1 with YAC cells for 4 h (NK cell activation) or with 2 μM P_261–269_ for 6 h (CD8^+^ T-cell stimulation) in 100 μl RPMI medium containing 10% FCS, glutamax, antibiotics and 30 μM β-mercaptoethanol, and 10 μM monensin and then stained as described [34]. Cells were analyzed on a FACS Calibur or Canto II (BD Biosciences) using FlowJo software (Tree Star).

### 2.4 Preparation of peptide-loaded bone marrow (BM)-DC and FI-PVM

Mouse BM-DC were expanded for 6 days in RPMI medium with 15% GM-CSF (culture supernatant of X63Ag cells), activated overnight with 100 ng/ml LPS and then pulsed for 1 h with 2 μM P_261–269_. Mice were immunized intravenously (i.v.) with 5 × 10^6^ peptide-loaded BM-DC in 200 μl PBS. FI-PVM was prepared as described [6] and was administered in 100 μl s.c. Mice were infected with PVM, 3–5 weeks after immunization.

### 2.5 Quantitative real-time PCR

Total lung RNA was purified using TRIzol (Invitrogen) and cDNA was transcribed (iScript cDNA Synthesis Kit; Bio-Rad Laboratories). PVM_SH_ RT-PCR was performed as described [35] in an iCycler (Bio-Rad Laboratories), 95 °C for 10 min and then 45 cycles of 95 °C for 15 s and 60 °C for 60 s. Copy numbers per lung were calculated from a standard curve generated using serially diluted PVM-SH cDNA. RT-PCR for IL-4, IFNγ and GAPDH were performed using the TaqMan Gene Expression Assays (Applied Biosystems) Mm00445259, Mm00801778 and Mm99999915. Relative expression of IL-4 and IFNγ normalized against GAPDH were calculated using a fixed point of the standard curve as calibrator.

### 2.6 Multiplex bead-based assay

To quantify IL-4 and IFNγ, fluoresceinated microbeads coated with capture antibodies (IL-4: BVD-1D11; IFN-γ:AN-18) were added to 50 μl BAL fluid and incubated overnight at 4 °C. Cytokines were detected with biotinylated anti-IFNγ (XMG1.2) and -IL-4 (BVD6-24G2), and PE-labeled streptavidin. Fluorescence was measured using a Luminex model 100 XYP (Luminex, Austin, TX, USA). Antibodies were purchased from BD Biosciences.

### 2.7 Adoptive transfer of CD8^+^ T-cells

Naïve and PVM-infected (d. 14 p.i.) donor mice were sacrificed, single cell suspensions prepared of lungs, spleens and MLNs were mixed and stained with PE-labeled antibodies against CD19, CD4, MHC-II and NKp46 (without Fc-block). Negative selection was performed using a BD Influx (BD Biosciences). Recipient mice received 5 × 10^6^ enriched cells in 200 μl PBS i.v., and then were infected with PVM.

## 3 Results

### 3.1 Dynamics of CD8^+^ T-cell responses in PVM-infected mice

**Fig. 1:**
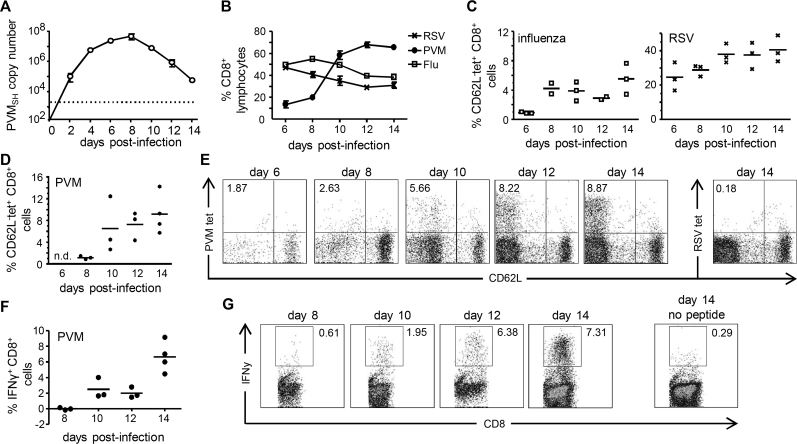
CD8^+^ T-cell kinetics in PVM and influenza-infected mice. BALB/c mice were infected i.n. with approximately 25 pfu PVM or 1 × 10^5^ EID_50_ influenza A/HK-x31 and sacrificed at the indicated days p.i. (A) PVM virus titers in the right lung determined by quantitative RT-PCR and converted to PVM-SH gene copy numbers per lung. The dotted line indicates the detection limit. Results are shown as mean ± SEM with 3 mice per group. (B) Frequency of total CD8^+^ T-cells as percentage of lymphocytes in the BAL determined by flow cytometry at the indicated days p.i. Results are shown as mean ± SEM with 3 mice per group. (C) Frequencies of virus-specific CD8^+^ T-cells in the BAL were determined by staining with MHC class I tetramers loaded with NP_147–155_ (influenza tetramer) or M2_82–290_ (hRSV tetramer), and tetramers loaded with unrelated peptides were used to measure background staining. The percentage of virus-specific CD8^+^ T-cells in the BAL of influenza (left graph) or hRSV (right graph) infected mice (CD62L^−^tetramer^+^) are shown after subtraction of background staining. (D and E) Frequencies of virus-specific CD8^+^ T-cells in the BAL of PVM infected mice, determined as described in (C) with P_261–269_-peptide loaded MHC class I tetramers. (D) Graph showing the results of individual mice and (E) representative FACS plots (gated on CD8^+^ cells) show the percentage of PVM tetramer+ (tet^+^) or hRSV tetramer^+^ CD62L^−^ cells at the indicated days p.i. (F, G) BAL cells from PVM-infected mice were restimulated *ex vivo* for 6 h in the presence of monensin with or without P_261–269_ peptide. (F) Corresponding graphs showing the results of individual mice (background frequencies in the absence of peptide are subtracted) and (G), representative FACS plots showing frequencies of IFNγ^+^ CD8^+^ cells after peptide restimulation. Data are representative of two independent experiments. BAL of uninfected mice did not contain any cells or detectable viral loads.

Intranasal infection with 25 pfu of PVM strain J3666 induced severe but sublethal disease in BALB/c mice, with weight reduction of approximately 15–20% of original body weight (data not shown). During the first days of infection, PVM rapidly replicated to high numbers (Fig. 1A). Viral copy numbers peaked at d. 8 p.i. and then declined.

In order to determine their protective capacity, we first studied CD8^+^ T-cell kinetics during primary PVM infection and compared these with the well-described CD8^+^ T-cell responses in influenza and hRSV-infected mice [36,37]. The relative proportions of CD8^+^ T-cells in the airways of PVM-infected mice strongly increased over time (Fig. 1B), and from d. 10 onwards approximately 60% of lymphocytes in the BAL were CD8^+^ T-cells. In influenza- and hRSV-infected mice, initially, the proportions of CD8^+^ T-cells in the airways were higher than in PVM-infected mice but then dropped, when relative proportions of CD8^+^ T-cells in PVM-infected mice were still rising (Fig. 1B). Quantification of virus-specific CD8^+^ T-cells with MHC class I tetramers containing a dominant epitope of either PVM (P_261–269_ [30]), influenza (NP_147–155_ [38]) or hRSV (M2_82–90_ [39]), demonstrated that NP_147–155_- and M2_82–90_-specific CD8^+^ T-cells were detectable at d. 6 p.i. and expanded until d. 8–10 p.i. when a plateau was reached (Fig. 1C). In PVM-infected mice, the BAL did not contain any P_261–269_-specific CD8^+^ T-cells at d. 6 p.i, and only a small population of P_261–269_-specific CD8^+^ T-cells could be detected at d. 8 p.i. (Fig. 1D and E). The relative proportions of P_261–269_ tetramer^+^ CD8^+^ T-cells further increased until d. 10 p.i. after which levels remained high (Fig. 1D and E). To determine whether PVM-specific CD8^+^ T-cell were functional, we quantified IFNγ production in virus-specific CD8^+^ T-cells after *ex vivo* P_261–269_ stimulation. Consistent with earlier publications [30,37], we found that IFNγ producing P_261–269_-specific CD8^+^ T-cells were barely detectable at d. 8 of infection (Fig. 1F and G) but then increased in numbers. At any time point of infection, the relative proportions of IFNγ-producing P_261–269_-specific CD8^+^ T-cells remained lower than that of P_261–269_ tetramer-stained cells. From these data we conclude that high proportions of CD8^+^ T-cells migrate to the lungs of PVM infected mice and that the appearance of virus-specific CD8^+^ T-cells in the airways is slightly delayed compared to influenza virus- or hRSV-infected mice.

### 3.2 Dynamics of innate responses to PVM infection

**Fig. 2:**
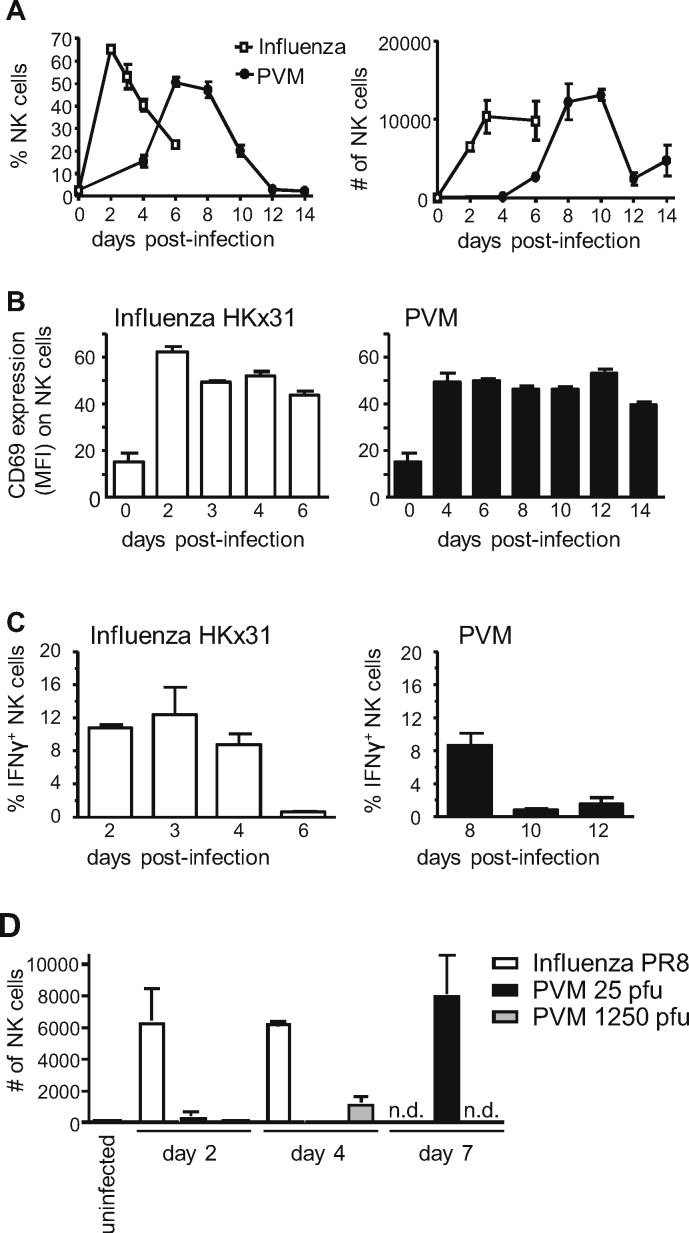
NK cell responses in PVM-infected mice compared to influenza-infected mice. BALB/c mice were infected i.n. with approximately 25 pfu PVM or 1 × 10^5^ EID_50_ influenza A/HK-x31 and sacrificed at the indicated days p.i. (A) NK cells (TCRβ^−^DX5^+^) as percentage of total lymphocytes (left panel) or in absolute numbers (right panel) in the BAL, as determined by flow cytometry. (B) Mean fluorescence intensities (MFI) of CD69 expression on NK cells in the BAL. (C) Percentage of IFNγ producing NK cells in the BAL after *ex vivo* restimulation with YAC cells (1:1) in the presence of monensin for 4 h. (D) Mice were infected with 150 EID_50_ influenza PR8, 25 pfu PVM (normal dose) or 1250 pfu PVM (high dose) and absolute numbers of NK cells (DX5^+^NKp46^+^TCRβ^−^) in the BAL were determined. Results are shown as mean ± SEM for 3 mice per group. n.d., not determined.

As PVM-specific CD8^+^ T-cells migrated relatively late to the lungs of PVM infected mice, we wondered whether migration of other immune cells was delayed also. Quantification of NK cells in the BAL demonstrated a prominent influx of NK cells into the airways of PVM-infected mice at d. 6 of infection, when approximately 50% of total infiltrating lymphocytes were NK cells (Fig. 2A, left panel). In absolute numbers (Fig. 1A, right panel) NK cell responses in PVM-infected mice peaked between days 8 and 10 of infection and then declined. In comparison, in the airways of influenza strain HKx31-infected mice (Fig. 1A) a large influx of NK cells, representing approximately 60% of total lymphocytes, was detected already at d. 2 p.i. with absolute numbers of infiltrating NK cells peaking at d. 3 of infection. Similar results were obtained in analyses of the BAL of hRSV-infected mice (Supplemenary Fig. 1). Both in influenza- and in PVM-infected mice, BAL NK cells displayed an activated phenotype (high CD69) and produced IFNγ upon stimulation *ex vivo* (Fig. 2B and C), indicating that they were functional. Thus, PVM-infected mice show a marked influx of NK cells into the airways, although at a later time point than in mice infected with influenza or hRSV.

PVM is a natural mouse pathogen and, unlike in case of HKx31, only a few viral particles suffice to establish severe disease in mice. To determine whether the low numbers of infecting virus particles explains for the shifted kinetics of NK cell responses in PVM compared to HKx31-infected mice, NK cell influx into the airways of PVM-infected mice was compared to that in mice infected with the mouse-adapted influenza strain PR8, which is more virulent than HKx31 and therefore used at 100–1000 fold lower concentration. Still, like HKx31, infection with PR8 (150 EID_50_) induced a prominent early NK cell influx into the airways (Fig. 2D, d. 2 and 4 p.i). Conversely, mice infected with a high dose of PVM (1250 pfu) lacked NK cells in the BAL at d. 2 p.i., and only minor numbers of NK cells were detected at d. 4 p.i. (Fig. 2D). In conclusion, both CD8^+^ T-cells and NK cells migrate to the BAL at a much later time point following infection with PVM than with influenza. The relatively late influx of NK cells into the airways of PVM-infected mice is likely to be explained by specific properties of this pneumovirus rather than by the low numbers of viral particles administered to cause infection.

### 3.3 P_261–269_-specific memory CD8^+^ T-cells provide partial protection against PVM-induced disease

**Fig. 3:**
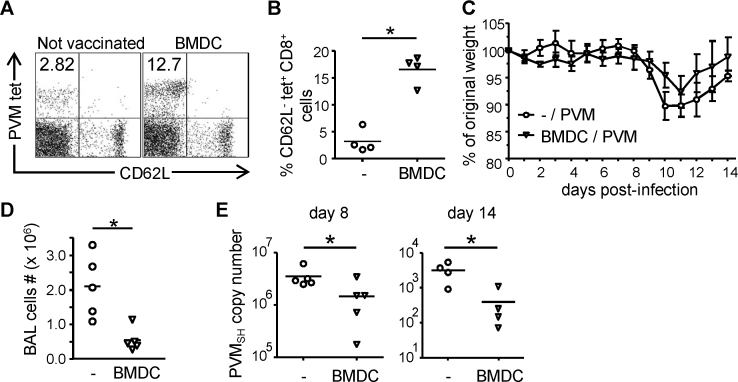
Effects of DCp immunization on control of PVM infection. Mice were immunized i.v. with 5 × 10^6^ P_261–269_-loaded BM-DCs or left untreated, and infected i.n. with approximately 15 pfu PVM 3–5 weeks later. 4–5 mice per group were sacrificed on d. 8 and 14 after PVM infection. (A and B) Frequency of P_261–269_-specific CD8^+^ cells in the BAL at d. 14 p.i. determined by tetramer staining as described in the legend to Fig. 2. (A) Representative FACS plots (gated on CD8^+^ cells), and (B) corresponding graphs showing results for individual mice. (C) Body weight of individual mice as a percentage of their initial weight. (D) Total numbers of cells in the BAL at d. 8 p.i. (E) Virus titer in the right lung determined by quantitative RT-PCR and converted to PVM-SH gene copy numbers. Data are representative of two independent experiments. Statistical analysis was performed using a Mann–Whitney *U*-test. *p < 0.05.

It has been shown that in PVM-infected mice, T-cells are responsible for viral clearance, but are also involved in immunopathology [31]. To determine whether PVM-specific memory CD8^+^ T-cells may confer immune protection, mice were immunized with GM-CSF-expanded BM-DC loaded with synthetic P_261–269_ (DCp) and then challenged with PVM. As shown in Fig. 3A and B, numbers of P_261–269_-specific CD8^+^ T-cells detected in the BAL of immunized mice were substantially higher than in non-immunized controls (Fig. 3A and B). Over the duration of the infection, DCp-primed mice lost less weight (Fig. 3C), displayed significantly reduced total-cell influx in the BAL (Fig. 3D), viral loads were significantly lower than in non-immunized mice (Fig. 3E), and peribronchial and interstitial cellular infiltrates were reduced (Supplementary Fig. 2), indicating an enhanced control of disease and viral loads.

**Fig. 4:**
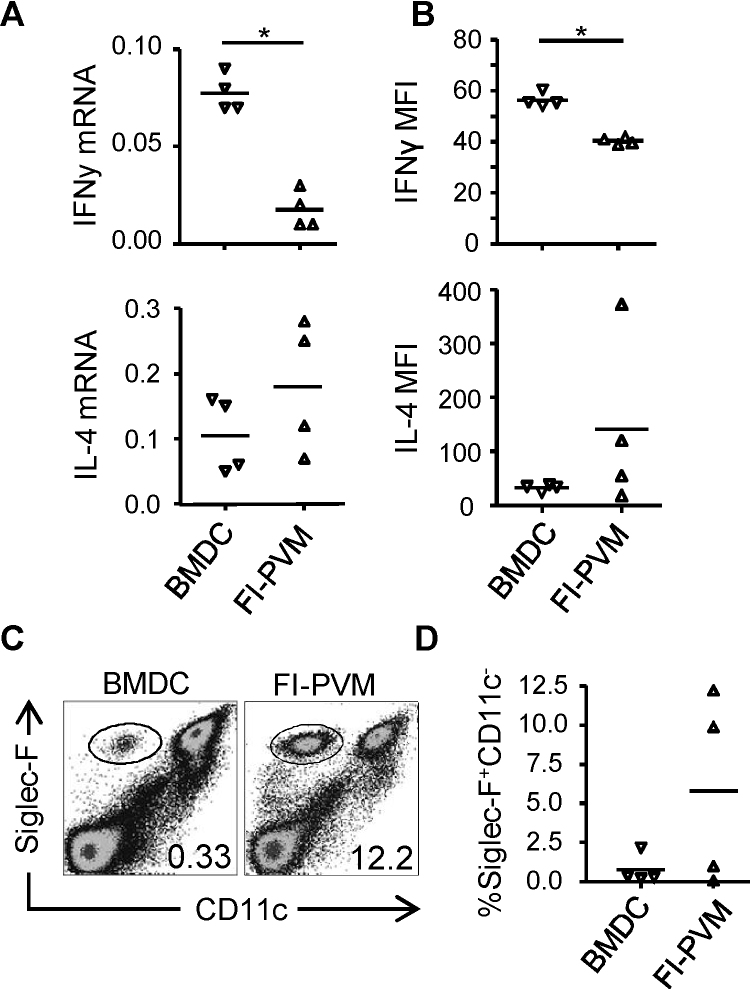
Cytokine production and eosinophils in the airways of PVM-infected, DCp-compared to FI-PVM-immunized mice. 4–5 mice per group were immunized i.v. with 5 × 10^6^ P_261–269_-loaded BM-DCs, or s.c. with FI-PVM, and infected i.n. with approximately 15 pfu PVM 3–5 weeks later. (A) Relative expression of IFNγ (upper graph) or IL-4 (lower graph) mRNA in the lungs 5 days after PVM infection, determined by Q RT-PCR. (B) Levels of IFNγ (upper graph) and IL-4 (lower graph) in the BAL fluid on d. 5 p.i., determined by luminex. (C) FACS plots showing an example of CD11c and Siglec-F staining on BAL cells of immunized mice 14 days after PVM infection. Gated samples were eosinophils (CD11c^−^Siglec-F^+^) as described [42]. (D) Frequency of eosinophils in the BAL of individual immunized mice 14 days after PVM infection, determined by flow cytometry. Statistical analysis was performed using a Mann–Whitney *U*-test. *p < 0.05.

Since vaccination with FI-PVM elicits an enhanced Th2 response upon PVM infection [40], we investigated the effect of DCp immunization on CD4 T-cell differentiation during PVM challenge. Compared with FI-PVM-immunized controls, mice immunized with P_261–269_-loaded DC displayed elevated amounts of IFNγ mRNA and cytokine levels in the lungs following challenge, indicating that they had developed a Th1-skewed immune response (Fig. 4A and B; upper panels). In contrast, FI-PVM immunized mice developed a Th2-skewed response, as indicated by the relatively high levels of IL-4 in the lungs (Fig. 4A and B; lower panels) and eosinophilia in two out of four mice (Fig. 4C and D). Thus, the presence of memory CD8^+^ T-cells specific for a single PVM-epitope led to enhanced control of virus replication and prevented Th2 skewing of PVM-induced CD4 T-cell responses upon PVM challenge, leading to a reduction of PVM-induced disease.

### 3.4 Protection conferred by adoptively transferred PVM-specific CD8^+^ T-cells

**Fig. 5:**
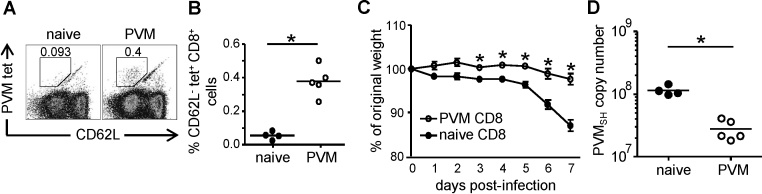
Effects of CD8^+^ T-cell transfer on PVM infection. CD8^+^ T-cells enriched from pooled lung-, spleen- and MLN-cells of PVM-infected (d. 14 p.i.) or naïve donor mice were transferred i.v. into recipient mice that were subsequently infected with approximately 25 pfu PVM and sacrificed at d. 7 p.i. (A) Representative FACS plots of gated CD8^+^ cells showing P_261–269_-specific T cells (CD62L^−^PVM tet^+^) in the lungs of mice that received CD8^+^ T-cells of PVM-infected (right) or naïve (left) donors. (B) Frequencies of P_261–269_-specific CD8^+^ cells in the lungs, determined by tetramer staining. (C) Body weight of individual mice as percentage of their initial weight at the indicated days p.i. (D) Virus titer in the right lung determined by Q RT-PCR and converted to PVM-SH copy numbers. Statistical analysis was performed using a Mann–Whitney *U*-test. *p < 0.05.

Since immunization with P_261–269_-loaded DC provided partial protection, we decided to assess the protective capacity of the total PVM-specific CD8^+^ T-cell response, targeting multiple epitopes. A mix of CD8^+^ T-cells enriched from the spleen, MLN and lungs of PVM-infected or uninfected mice were adoptively transferred into recipient mice that then were infected with PVM. At d. 7 p.i. a clear population of P_261–269_-tetramer^+^ cells was detectable in the lungs of mice that had received CD8^+^ T-cells of PVM-infected donors, but not in the lungs of recipients that had received naïve CD8^+^ T-cells of uninfected controls (Fig. 5A and B). In addition, recipients receiving immune cells from infected mice showed significantly reduced weight-loss and viral load (Fig. 5C and D). These results show that PVM-specific CD8^+^ T-cells, despite being a major cause of pathology in pneumovirus infections, can provide protection against PVM infection.

## 4 Discussion

Despite the fact that hRSV is a major cause of disease in infants, there still are major gaps in our knowledge of the host response against this virus. There is an increasing interest in using the natural mouse pathogen PVM to mimic and study severe pneumovirus infections. We have used this model to study the role of CD8^+^ T-cells in conferring protection against disease. Influx of both NK and CD8^+^ T-cells into the BAL of PVM-infected mice was markedly delayed compared to that in mice infected with influenza or hRSV (Figs. 1 and 2). However, from d. 10 p.i. onwards, extremely high numbers of CD8^+^ T-cells were present in the airways of PVM-infected mice, coinciding with disease. The relatively late immune activation seen in the PVM-infected mice was not explained by the quantities of administered viral particles, as both sublethal and lethal doses of PVM failed to induce an early NK cell influx in the infected respiratory tissue (Fig. 1), whereas both high dose HKx31 and low dose PR8 (representing comparable ID50s) caused an early NK cell influx, well detectable at d. 2 p.i. If not the quantities of administered particles, differing replication kinetics may explain the differences in kinetics of immune activation between PVM and influenza infection, although it should be noted that PVM rapidly replicates during the first days of infection, reaching titers of approximately 10^5^ particles/lung at d. 2 p.i. (Fig. 1). Alternatively, the relatively late influx of lymphocytes into the airways of PVM-infected mice is consistent also with recent observations that the nonstructural proteins of PVM (NS1 and NS2) inhibit type I and type III interferon responses [27,28]. In these studies, inflammation in the airways of PVM-infected mice was found to be still limited at d. 3 p.i., while at d. 6 p.i., high levels of chemokines and cytokines such as MCP-1, RANTES, MIP-1α and IL-15 were produced [27,28]. These chemokines are likely to attract NK cells to the airways, as well as CD8^+^ T-cells [31].

The finding that CD8^+^ T-cells cause pathology in the PVM-mouse model [31] has raised questions about the use of a vaccine designed to stimulate a pneumovirus-specific CD8^+^ T-cell response. However, we show that mice immunized with BM-DCs pulsed with PVM P_261–269_ displayed a Th1-skewed immune response and reduced viral loads following challenge (Figs. 3 and 4), suggesting that vaccine-induced CD8^+^ T-cell memory protects against pneumovirus-induced disease. In an earlier study [41], immunization with PVM P_261–269_ in IFA was unsuccessful in protecting mice against PVM-infection unless co-administered with a PVM-derived CD4 T-cell epitope. Interestingly, the peptide/IFA immunization protocol used in that study resulted in mixed Th1/Th2 responses to the included CD4 T-cell epitope, in contrast to the Th1 responses observed in PVM-challenged DCp-immunized mice (Fig. 3). Thus, immunization-induced PVM-specific memory CD8^+^ T-cells protect against PVM-associated disease, but the degree of protection and effects of immunization on CD4 T-cell differentiation depend on the strategy for epitope delivery and used adjuvant. Importantly, transfer of CD8^+^ T-cells isolated from PVM-infected mice, which are targeted to a broad range of epitopes, almost entirely abrogated weight-loss in recipients and significantly reduced viral loads following challenge with PVM (Fig. 5). Taken together, the data presented here demonstrate that the presence of already primed PVM-specific CD8^+^ T-cells at the time point of PVM-infection leads to enhanced control of viral loads and prevents T-cell-driven immunopathology.

In conclusion, we have shown PVM-specific CD8^+^ T-cells provide partial protection against PVM-induced disease, probably by preventing Th2 skewing of PVM-specific immune responses and by early control of viral loads. Our findings strongly suggest that pneumovirus vaccines designed to induce antigen-specific CD8^+^ T-cell memory may offer effective protection against pneumovirus-induced disease.

## Appendix A Supplementary data

Supplementary Fig. 1 NK cell responses in PVM-compared to hRSV-infected mice. BALB/c mice were infected i.n. with approximately 25 pfu PVM or 5 × 10^6^ pfu hRSV and sacrificed at the indicated days p.i. NK cells (TCRβ^−^DX5^+^) are shown as percentage of total lymphocytes as determined by flow cytometry.

Supplementary Fig. 2 Pre-existing PVM-specific CD8^+^ T cells diminish severe cellular infiltration upon intranasal PVM infection. BALB/c mice were immunized i.v. with P_261–269_-loaded BM-DCs (A) or left untreated (B), and infected i.n. with 15 pfu of PVM 5 weeks later. The mice were sacrificed 9 days after PVM infection by i.p. injection of sodium pentobarbital, the lungs were slightly inflated transtracheally using 500 (l 4% phosphate-buffered formalin, removed, fixed in 4% phosphate-buffered formalin, paraffin-embedded, sectioned and stained with hematoxylin and eosin. Two photos (upper panel, 10× objective; lower panel, 40× objective) of one mouse per group are shown.
