# Supplementary material for: Pre-existing virus-specific CD8+ T-cells provide protection against pneumovirus-induced disease in mice
Source: Vaccine. 2012 Oct 5;30(45):6382–8. doi: 10.1016/j.vaccine.2012.08.027 (PMC3465553; doi:10.1016/j.vaccine.2012.08.027)
Supplement: Supplementary Fig. 1 — NK cell responses in PVM-compared to hRSV-infected mice. BALB/c mice were infected i.n. with approximately 25 pfu PVM or 5 × 10^6^ pfu hRSV and sacrificed at the indicated days p.i. NK cells (TCRβ−DX5+) are shown as percentage of total lymphocytes as determined by flow cytometry. [file mmc1.ppt]

## Slide 1
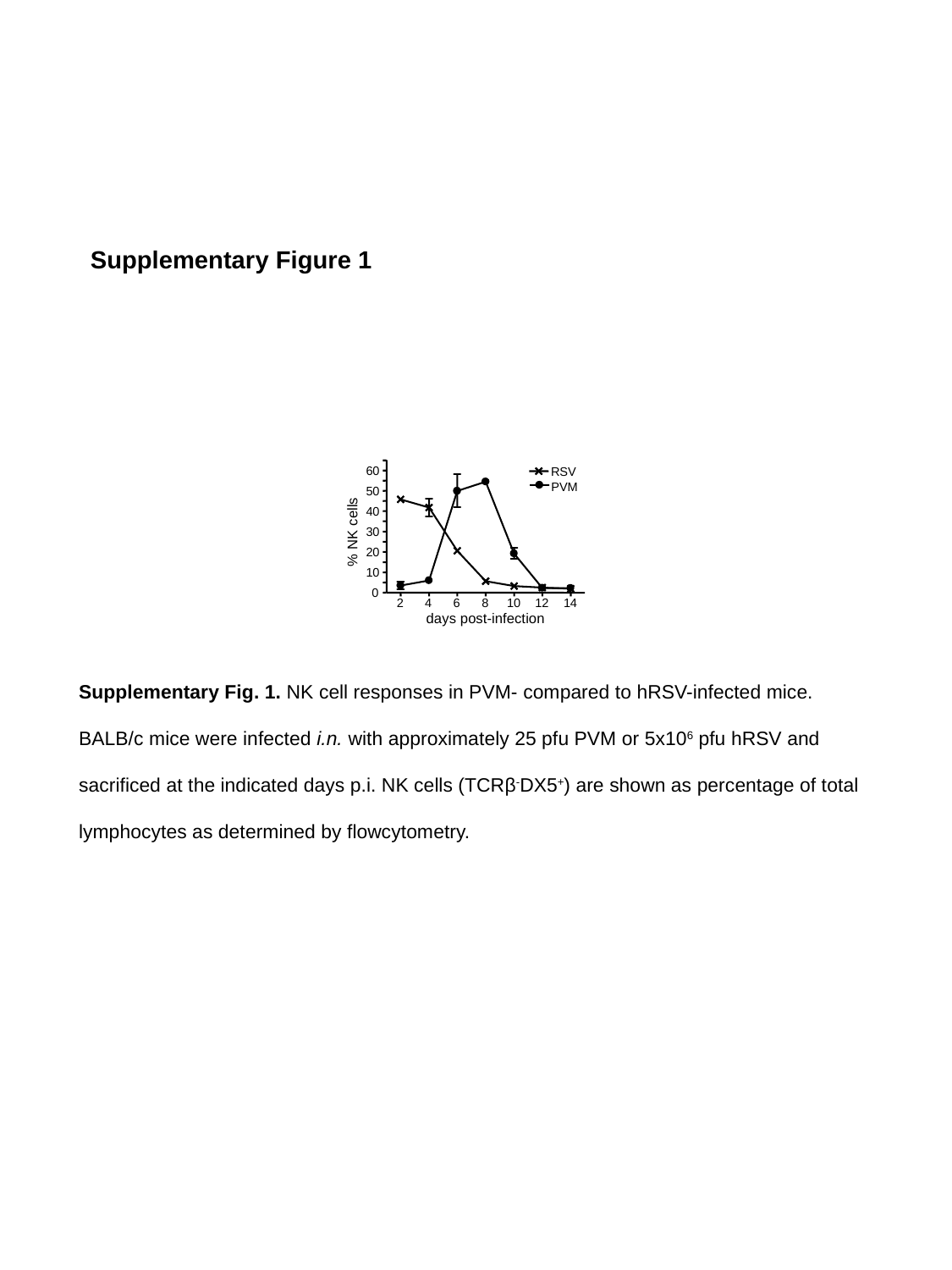

Supplementary Figure 1
60
RSV
PVM
50
40
% NK cells
30
20
10
0
2
4
6
8
10
12
14
days post-infection
Supplementary Fig. 1. NK cell responses in PVM- compared to hRSV-infected mice. BALB/c mice were infected i.n. with approximately 25 pfu PVM or 5x10^6^ pfu hRSV and sacrificed at the indicated days p.i. NK cells (TCRβ-DX5+) are shown as percentage of total lymphocytes as determined by flowcytometry.
