# Supplementary material for: Pre-existing virus-specific CD8+ T-cells provide protection against pneumovirus-induced disease in mice
Source: Vaccine. 2012 Oct 5;30(45):6382–8. doi: 10.1016/j.vaccine.2012.08.027 (PMC3465553; doi:10.1016/j.vaccine.2012.08.027)
Supplement: Supplementary Fig. 2 — Pre-existing PVM-specific CD8+ T cells diminish severe cellular infiltration upon intranasal PVM infection. BALB/c mice were immunized i.v. with P261–269-loaded BM-DCs (A) or left untreated (B), and infected i.n. with 15 pfu of PVM 5 weeks later. The mice were sacrificed 9 days after PVM infection by i.p. injection of sodium pentobarbital, the lungs were slightly inflated transtracheally using 500 (l 4% phosphate-buffered formalin, removed, fixed in 4% phosphate-buffered formalin, paraffin-embedded, sectioned and stained with hematoxylin and eosin. Two photos (upper panel, 10× objective; lower panel, 40× objective) of one mouse per group are shown. [file mmc2.ppt]

## Slide 1
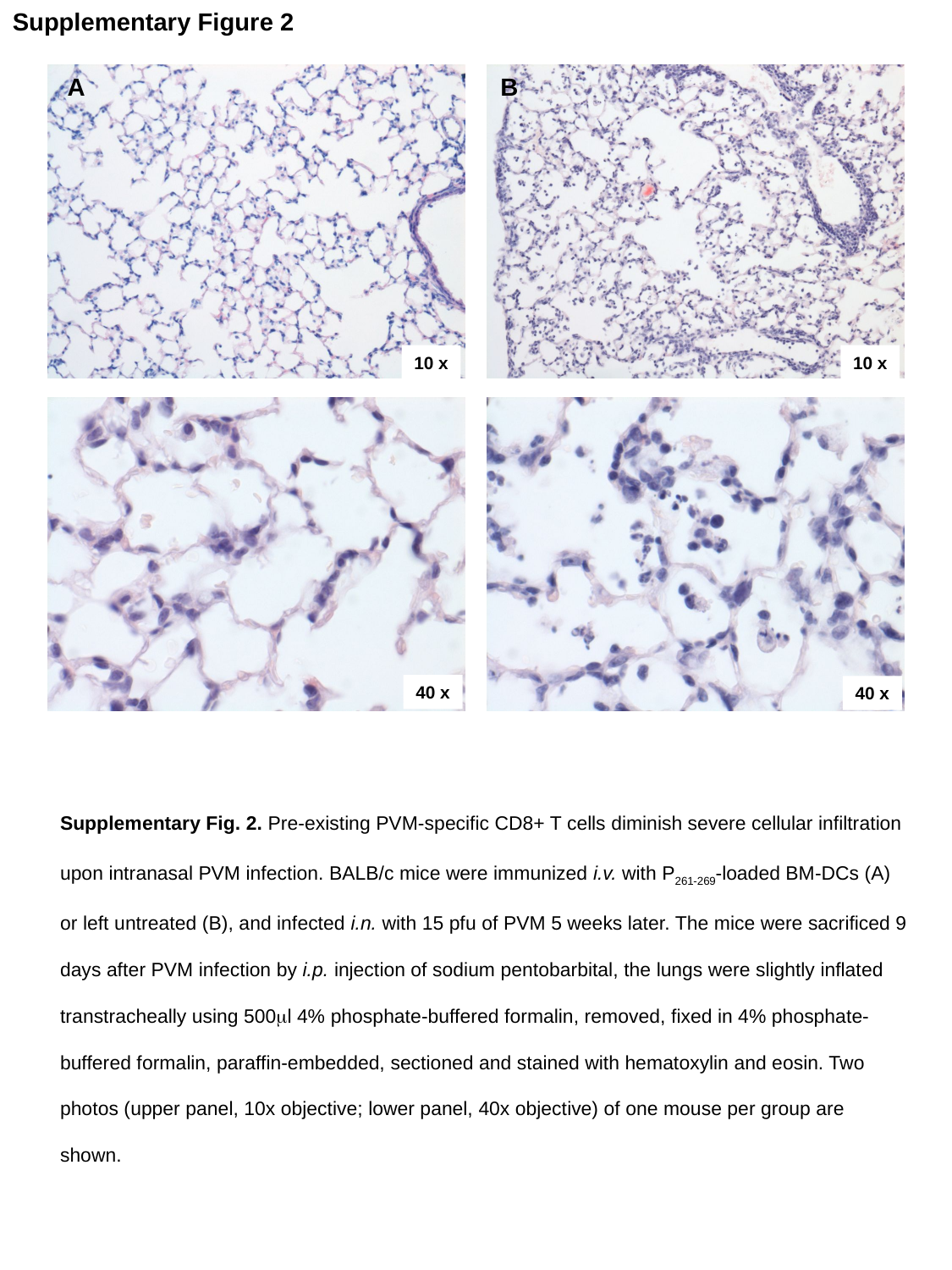

Supplementary Figure 2
A
B
10 x
10 x
40 x
40 x
Supplementary Fig. 2. Pre-existing PVM-specific CD8+ T cells diminish severe cellular infiltration upon intranasal PVM infection. BALB/c mice were immunized i.v. with P261-269-loaded BM-DCs (A) or left untreated (B), and infected i.n. with 15 pfu of PVM 5 weeks later. The mice were sacrificed 9 days after PVM infection by i.p. injection of sodium pentobarbital, the lungs were slightly inflated transtracheally using 500μl 4% phosphate-buffered formalin, removed, fixed in 4% phosphate-buffered formalin, paraffin-embedded, sectioned and stained with hematoxylin and eosin. Two photos (upper panel, 10x objective; lower panel, 40x objective) of one mouse per group are shown.
